# *Laccase2*-Mediated Mandibular Development Is Crucial for Larval Feeding Behavior in the Silkworm, *Bombyx mori*

**DOI:** 10.3390/insects17090893

**Published:** 2026-08-25

**Authors:** Dalin Zhu, Chenxin Sun, Yutong Liu, Ling Ye, Qingyun He, Mengying Fang, Bin Lin, Anjiang Tan, Xiaoxiao Ji

**Affiliations:** 1 Jiangsu Key Laboratory of Sericultural and Animal Biotechnology, School of Biotechnology, Jiangsu University of Science and Technology, Zhenjiang 212100, China; zdlsri@stu.just.edu.cn (D.Z.); sunchenxin@petalmail.com (C.S.); liuyutong010203@163.com (Y.L.); 17855203627@163.com (L.Y.); 231111801115@stu.just.edu.cn (Q.H.); fangmengying0806@163.com (M.F.); just20230909@163.com (B.L.); atan@just.edu.cn (A.T.); 2 Key Laboratory of Silkworm and Mulberry Genetic Improvement, Ministry of Agriculture and Rural Affairs, Sericultural Scientific Research Center, Chinese Academy of Agricultural Sciences, Zhenjiang 212100, China

**Keywords:** *Bombyx mori*, *Laccase2*, mandibular development, CRISPR/Cas9, feeding behavior

## Abstract

The phenoloxidase gene *Laccase2* (*Lac2*) is an indispensable survival gene in insects, playing key roles in cuticle tanning, exoskeleton development and pigment deposition. However, in lepidopteran insects, the roles of *Lac2* in mandibular development and feeding behavior remain largely unexplored. In this study, we demonstrated that *Lac2* is essential for mandibular development in early larvae of the model lepidopteran, the silkworm, *Bombyx mori*. We cloned and characterized *BmLac2* and found that it is evolutionarily conserved in lepidopteran insects. Disruption of *BmLac2* via CRISPR/Cas9 system induced mandibular defects, which directly impaired larval feeding behavior and culminated in developmental arrest and larval mortality. Moreover, knockout of *BmLac2* led to dopamine accumulation during the dormancy stage, but the dopamine level returned to normal after molting, without altering feeding motivation in mutant larvae. Furthermore, RNA sequencing (RNA-seq) and quantitative real-time polymerase chain reaction (qRT-PCR) analyses revealed that loss of *BmLac2* affected the expression of genes associated with cuticular formation and immunity, also contributing to larval mortality alongside feeding defects. Altogether, *BmLac2*-mediated mandibular development and cuticular melanization play key roles in regulating feeding behavior and immunity in early-stage silkworm larvae, making this gene a potential target for early intervention against lepidopteran pests.

## Simple Summary

*Laccase2* (*Lac2*) is a conserved insect gene essential for cuticle formation, pigmentation, and survival. However, its potential roles in insect feeding remain poorly understood. In this study, we investigated the function of *Lac2* in the silkworm, *Bombyx mori*, using CRISPR/Cas9 system. Our findings suggest that loss of *BmLac2* disrupted larval mandibular development, resulting in abnormal feeding behavior, reduced food intake, developmental arrest, and ultimately larval mortality. Although *BmLac2* deficiency caused dopamine accumulation during the dormancy stage, dopamine levels recovered after molting and did not affect feeding motivation, suggesting that feeding impairment mainly resulted from defective mandibular development. These findings uncover a previously unrecognized role of *Lac2* in regulating mandibular development and feeding ability in lepidopteran larvae.

## 1. Introduction

In insects, cuticle tanning and melanization are important physiological processes that help form a strong exoskeleton supporting normal structure, preventing water loss, and protecting against pathogens [1]. *Lac2* has been recognized as an essential enzyme involved in these vital processes across different insects [2]. *Lac2* is a copper enzyme that catalyzes the oxidation of catecholamines, resulting in cross-link formation between cuticular components and the hardening of the insect cuticle [3]. Functional analysis revealed that *Lac2* dysfunction leads to cuticle thinning and defective pigmentation in various insect species, including *Tribolium castaneum*, *Drosophila melanogaster*, *Bombyx mori*, *Aedes albopictus*, *Monochamus alternatus* and *Nilaparvata lugens* [4,5,6,7,8,9,10]. Knocking down *Lac2* in *T. castaneum* during the larval stage causes shorter larvae, lighter body color, failed molting, and death. *Lac2* knockdown during the pupal stage lightens the body color of both pupae and adults and impairs the adult host defense against fungi [4,5]. In *D. melanogaster*, knockdown of *Lac2* impairs melanization and prevents the development of normal wing cuticles and wing sensory bristles [6]. In addition to cuticle tanning and pigmentation, *Lac2* also contributes to eggshell hardening. In *A. albopictus*, *Lac2* knockdown prevents pigmentation and sclerotization of mosquito eggshells, resulting in colorless, deformed eggs that enlarge or break and later die in the water [8]. *Lac2* knockout in *Acyrthosiphon pisum* results in colorless eggs with weaker shells that are more susceptible to fungal infections and fail to hatch [11]. Therefore, *Lac2* is a core regulator governing the sclerotization and pigmentation of the cuticle and eggshell, which in turn confer immune defense on the insects. Its essential role in insect survival makes it a promising target for pest control.

Meanwhile, *Lac2* acts as a key metabolic enzyme in the dopamine signaling pathway. Dopamine is synthesized from the substrate L-tyrosine through a cascade of catalytic reactions mediated by *tyrosine hydroxylase* (*TH*) and *dopa decarboxylase* (*DDC*). The metabolism of dopamine is catalyzed by *Lac2*, *arylalkylamine-N-acetyltransferases* (*aaNat*), and *N-β-alanyl dopamine synthetase* (*ebony*) [12,13,14,15]. *Lac2* catalyzes the oxidation of dopamine and its precursor L-dopa, converting them into quinones that enter the melanin synthesis pathway, thereby regulating dopamine levels. The stability of the dopamine signaling pathway is essential for insects to maintain normal physiological functions, whereas disruption of dopamine signaling can severely impair multiple vital processes, including feeding, locomotion, mating, and social behaviors [16,17,18,19]. In honey bees, a dopaminergic system drives foraging motivation. Head dopamine levels and waggle dancing decrease following negative events such as predator attacks, but can be restored by elevating brain dopamine [20]. However, it remains unclear whether *Lac2*, as a key regulator of dopamine metabolism, also plays a role in regulating feeding behavior. Although *Lac2* has been extensively studied through RNA interference (RNAi) in many insects, its functions in lepidopterans remain largely unexplored due to RNAi inefficiency in this order.

The silkworm, *B. mori*, is an insect of significant economic value and a well-known model in Lepidoptera [21,22]. In the present study, the *BmLac2* gene was cloned and characterized. The temporal expression pattern of *BmLac2* was also analyzed by qRT-PCR. A binary transgenic CRISPR/Cas9 system was used to generate *BmLac2* mutants. Our results revealed that no significant differences were observed between the *BmLac2* mutant and the wild-type before the 1st instar molting, whereas abnormal melanization and impaired feeding behavior phenotypes emerged in the mutants after the first ecdysis. To explore the underlying molecular mechanisms, RNA-seq and qRT-PCR analyses were further performed. Our findings demonstrated that in addition to its canonical function in cuticle melanization and immunity, *BmLac2* plays a critical role in maintaining feeding behavior by ensuring normal mandibular development in the early stage of the silkworm.

## 2. Materials and Methods

### 2.1. Silkworm Strain

Nistari, a multivoltine and non-diapausing silkworm strain fed on fresh mulberry leaves, was used for all experiments. They were raised in a standard environment with a room temperature of 25 °C and a relative humidity of 60~65% [23].

### 2.2. Bioinformatics Analysis

The amino acid sequences of *Lac2* from different insects were obtained from the NCBI (https://www.ncbi.nlm.nih.gov/) (accessed on 15 August 2023). SMART (https://smart.embl.de/) (accessed on 26 December 2025) was used for the prediction and analysis of protein structures. Amino acid sequences were aligned using Clustal X 2.0 [24]. A phylogenetic tree was constructed using the maximum likelihood approach within the MEGA 5.10 software package [25].

### 2.3. CRISPR/Cas9-Mediated Plasmid Construction and Genetic Transformation

To generate mutations in the *BmLac2* gene, we used a binary transgenic CRISPR/Cas9 system [26]. The HR5IE1-EGFP-nos-Cas9 (nos-Cas9) line of *B. mori* expressing Cas9 nuclease under nos promoter control had been maintained in our laboratory. The two single guide RNAs (sgRNAs) were designed based on the cDNA sequence of *BmLac2* and targeted the regions of exon 4 and exon 7, respectively. The plasmid [IE1-DsRed-U6-sgRNA] (U6-sgRNA) carrying two sgRNAs was constructed to express *BmLac2*-specific sgRNAs. To generate the U6-sgRNA line, the mixture of piggyBac helper plasmids and U6-sgRNA plasmids was microinjected into G0 embryos. G0 adults were sib-mated to produce G1 progeny, and the G1 positive line was chosen based on red fluorescence from DsRed expression (AZ100; Nikon, Tokyo, Japan) [27]. The U6-sgRNA line and nos-Cas9 line were crossed to obtain *BmLac2* mutants with dual fluorescence. The primers used for plasmid construction were listed in Table S1.

### 2.4. RNA Extraction and cDNA Synthesis

Total RNA was extracted from tissues of wild-type or mutant larvae using TRIzol Reagent (Ambion, Kaufungen, Germany). Then cDNA was synthesized from 1 μg of the total RNA using the PrimeScript™ RT Reagent Kit with gDNA Eraser (Takara Bio, Shiga, Japan) following the manufacturer’s instructions.

### 2.5. Quantitative Real-Time PCR (qRT-PCR)

Quantitative real-time PCR was conducted with the SYBR^TM^ Green Realtime PCR Master Mix (TOYOBO, Osaka, Japan) on the CFX Connect Real-Time PCR System (Bio-Rad, Hercules, CA, USA). The 20 μL reaction mixture contained 1 μL of cDNA template, 1 μL of paired primers (10 μM), 10 μL of SYBR™ Green mix, and 8 μL of Invitrogen™ UltraPure™ DNase/RNase-Free distilled water (Thermo Fisher Scientific, Waltham, MA, USA). The qPCR thermal cycling protocol was: 95 °C for 10 min, followed by 40 cycles of 95 °C for 15 s and 60 °C for 1 min. The *B. mori* ribosomal protein 49 (*Bmrp49*) was used as an internal reference. The 2^−ΔΔCt^ method was used to calculate the mRNA expression levels. The primers used were also listed in Table S1.

### 2.6. Genomic DNA Isolation and Mutagenesis Analysis

Genomic DNA extraction from Δ*Lac2* and wild-type (WT) larvae was carried out using the TransDirect^®^ Animal Tissue PCR Kit (TransGen Biotech, Haidian, Beijing, China) according to the manufacturer’s protocol. For mutation analysis, the genomic DNA was amplified by PCR using KOD Plus (Takara, Kusatsu, Shiga, Japan) and *BmLac2*-specific primers. The PCR products were gel-purified, cloned into the pJET-1.2 vector (Thermo Fisher Scientific, Waltham, MA, USA) and sequenced. The *BmLac2*-specific primers were listed in Table S1.

### 2.7. Body Weight Measurement

Thirty wild-type or mutant silkworms were reared separately for body weight measurement. Each genotype contained 3 biological replicates. Body weight was measured and recorded every 24 h from the time the larvae molted into the 2nd instar using an electronic analytical balance (Sartorius, Göttingen, Germany). Five larvae per group were measured to reduce the error.

### 2.8. Feeding Behavior Tests

For the feeding motivation test, wild-type silkworms and Δ*Lac2* mutants that had just molted into the 2nd instar without feeding were separately placed 5 cm away from mulberry leaves. The time taken by the test larvae to crawl toward the leaves and initiate feeding behavior was recorded. Each group contained 30 test larvae.

For the food intake test, chopped fresh mulberry leaves were evenly spread in sterile culture dishes, and each larva was placed individually on the leaf surface. When the larvae began feeding, the behavior was recorded using an industrial camera (MV-CS200-10UC, HIKROBOT, Hangzhou, China). The number of feeding bouts within 1 min was counted from the video, and changes in the area of mulberry leaves before and after larval feeding were measured using ImageJ (Java 1.8.0_322).

### 2.9. Scanning Electron Microscopy Analysis of Mandible Microstructure

Head tissues of silkworm larvae were excised at the late 1st instar (L1D3), immediately after molting into the 2nd instar without feeding (L2D0), 24 h post-feeding (L2D1), and 48 h post-feeding (L2D2). The excised heads were washed in pure water and fixed with 4% paraformaldehyde. The fixed samples were dehydrated via exposure to gradually increasing concentrations of ethyl alcohol (30%, 50%, 70%, 80%, and 90%) using a rotary machine. The heads were dried in a critical-point dryer and then coated with platinum prior to observation under a scanning electron microscope (Hitachi SU8100, Hitachi High-Technologies Corporation, Tokyo, Japan) [28,29].

### 2.10. Measurement of the DA Concentration

Silkworm larvae were frozen in liquid nitrogen for future use. Subsequently, 0.01 g of tissue was homogenized in 100 μL of 2.5% perchloric acid solution at 4 °C for 60 s at 60 Hz for 3 cycles, followed by ultrasonic treatment at 4 °C for 20 min. The homogenate was then centrifuged at 12,000 rpm for 20 min at 4 °C, and the supernatant was filtered through a 0.22 μm membrane filter prior to detection by the Agilent HPLC 1200 system (Agilent Technologies, Santa Clara, CA, USA). A GOLD-C18 (250 × 4.6 mm, 5 μm) was used. A 20 μL sample was eluted with the pH 3.06 buffer (containing 1 mmol/L sodium heptanesulfonate and 30 mmol/L sodium dihydrogen phosphate-phosphoric acid solution) and methanol (85:15, *v*/*v*) for isocratic elution at a flow rate of 1 mL/min at 40 °C [30,31].

### 2.11. RNA-Seq Analysis

Total RNA was extracted from the whole bodies of three unfed wild-type (WT) or Δ*Lac2* at 0 day of the 2nd larval instar (L2D0) immediately after ecdysis, respectively. Three biological replicates for each genotype were set for the experiment. The RNA-seq libraries were constructed from enriched and fragmented total RNA using the NEBNext^®^ Ultra™ RNA Library Prep Kit for Illumina^®^ (NEB, Ipswich, MA, USA) following the manufacturer’s instructions. High-throughput sequencing was subsequently performed on the Illumina NovaSeq 6000 platform (LianChuan Biological Company, Hangzhou, China). Raw sequencing data were subjected to quality control and filtration, followed by alignment to the reference genome of *B. mori* retrieved from the NCBI database (https://www.ncbi.nlm.nih.gov/, accessed on 15 August 2023). Differentially expressed genes (DEGs) identified from the comparison between the wild-type and Δ*Lac2* mutant strains were subjected to functional annotation against the Gene Ontology (GO) and Kyoto Encyclopedia of Genes and Genomes (KEGG) databases.

### 2.12. Statistical Analysis

All results are expressed as mean ± standard deviation (SD) based on three independent biological replicates. For two-group comparisons, statistical significance was determined by Student’s *t*-test (* *p* < 0.05; ** *p* < 0.01; *** *p* < 0.001; **** *p* < 0.0001). All statistical evaluations were conducted using GraphPad Prism 8 software (GraphPad Software, San Diego, CA, USA).

## 3. Results

### 3.1. Characterization and Expression Pattern of BmLac2

**Figure 1:**
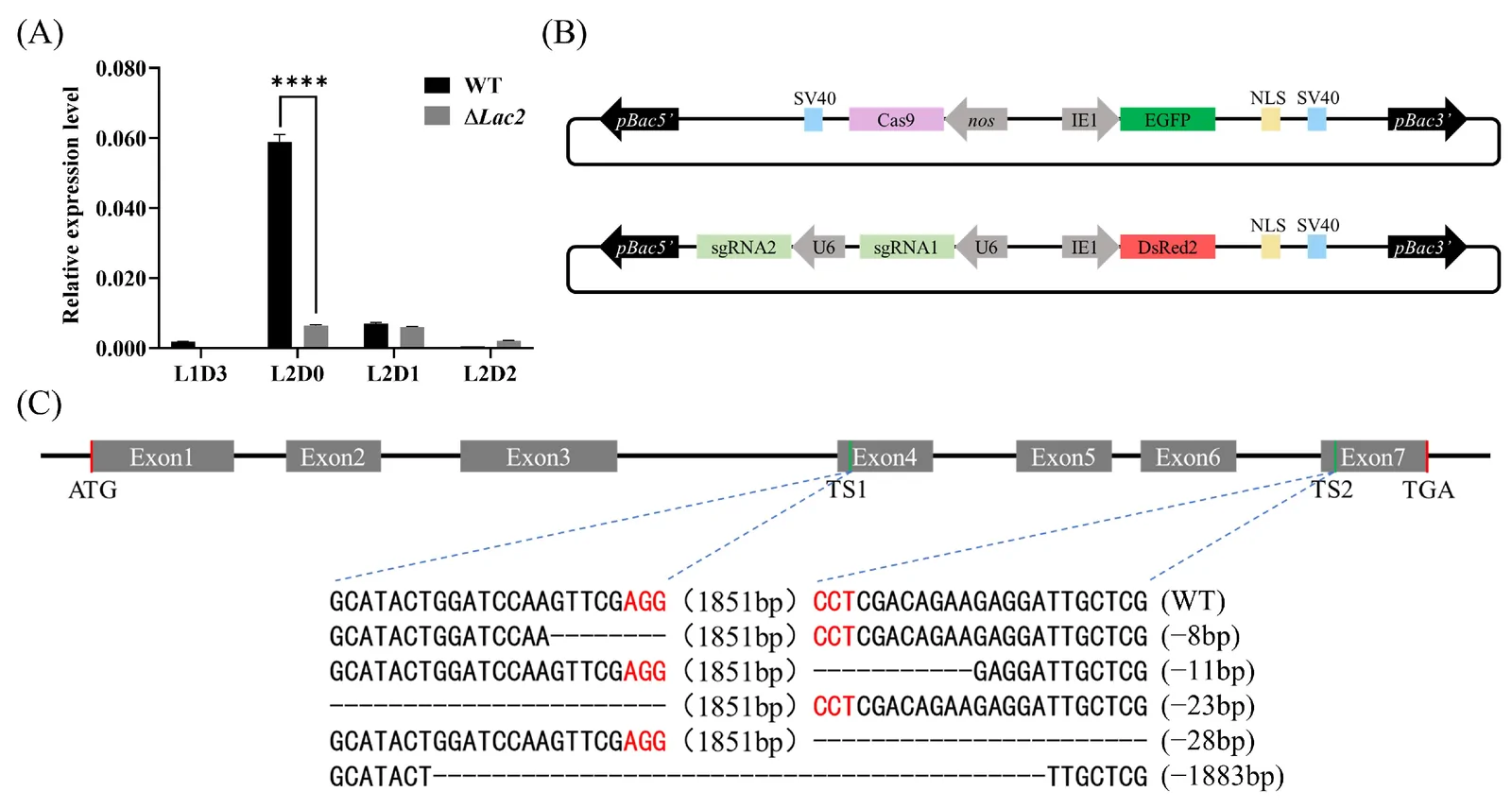
Generation of CRISPR/Cas9-mediated mutations. (**A**) Relative expression levels of *BmLac2* in WT and mutant (Δ*Lac2*) individuals before the first ecdysis (L1D3), during dormancy (L2D0), and after ecdysis (L2D1-L2D2). Error bars are ± standard deviation (SD), and statistical comparisons between two groups were performed using a two-tailed Student’s *t*-test (**** *p* < 0.0001). (**B**) Schematic representation of piggyBac-based transgenic plasmids construction. The expression of Cas9 was mediated by the nos promoter, and the expression of *BmLac2* sgRNAs was driven by the U6 promoter. (**C**) Schematic illustration of the sgRNAs targeting sites in *BmLac2* gene. Exons are shown as gray boxes, while introns are indicated by straight lines. The sgRNA target sites, TS1 and TS2, are situated on exons 4 and 7, respectively. Mutations of *BmLac2* in offspring after crossing nos-Cas9 and U6-sgRNAs transgenic silkworm lines were detected. ‘–’ represents the bases that are deleted. Red text represents the NGG PAM motif.

The *BmLac2* sequence contains a complete open reading frame of 2259 base pairs, encoding a protein of 752 amino acid residues. Prediction of protein domains was performed using the SMART website (https://smart.embl.de/) (accessed on 26 December 2025). Three conserved motifs, Cu-oxidase, Cu-oxidase_2 and Cu-oxidase_3, were presented (Figure S1A). The *B. mori Lac2* protein sequence was aligned with homologs from *T. castaneum*, *Sitophilus oryzae*, *Papilio machaon*, and *Manduca sexta* (Figure S1B). A phylogenetic tree was constructed using *Lac2* proteins from *B. mori* and other species (Figure S1C). These results revealed that BmLac2 exhibits high similarity to Lac2 proteins of other lepidopteran species except *M. sexta*, suggesting that these proteins are evolutionarily conserved and derived from a common ancestor. The temporal and spatial expression profiles of *BmLac2* were analyzed using quantitative real-time PCR. The results showed that on day L5D3 (Figure S1D), the *BmLac2* gene was not widely expressed, but was detected in the Malpighian tubules and testes. According to previous studies, *BmLac2* is highly expressed in the epidermis during the molting stage. Therefore, we detected its expression before and after molting at early larval stages (Figure 1A). The expression level of the *BmLac2* gene was extremely low before molting (L1D3), almost undetectable. However, its expression increased sharply during molting (L2D0), reaching nearly 33 times the pre-molting level. But immediately after molting (L2D1), the expression level of *BmLac2* decreased rapidly and remained low at the following stage (L2D2). These results demonstrated that *BmLac2* expression was regulated in a molting-dependent manner, with peak expression during molting and a rapid decline thereafter, which is likely related to new cuticle formation.

### 3.2. Construction of BmLac2 Mutant with the CRISPR/Cas9 System

A binary transgenic CRISPR/Cas9 system was conducted according to our previous methods. Within this experimental system, the expression of Cas9 protein is controlled by the nos promoter, and sgRNA expression is directed by the U6 promoter derived from silkworms (Figure 1B). *BmLac2* contains seven exons, and the sgRNA target sites were designed on the exon 4 and exon 7 (Figure 1C). Genomic DNA was isolated from the progeny of crosses between the nos-Cas9 and U6-sgRNA strains to characterize deletion mutations at the two target sites of *BmLac2*. Multiple distinct deletion mutations were detected in these mutants (Figure 1C). Furthermore, qRT-PCR was conducted to quantify the mRNA expression differences in *BmLac2* between wild-type and mutant strains. The results showed that knockout of *BmLac2* reduced *BmLac2* transcript levels by 89.13% in mutants compared with wild-type individuals at the 1st-instar molting stage (Figure 1A).

### 3.3. Loss of BmLac2 Resulted in Larval Developmental Arrest and Impaired Pigmentation

**Figure 2:**
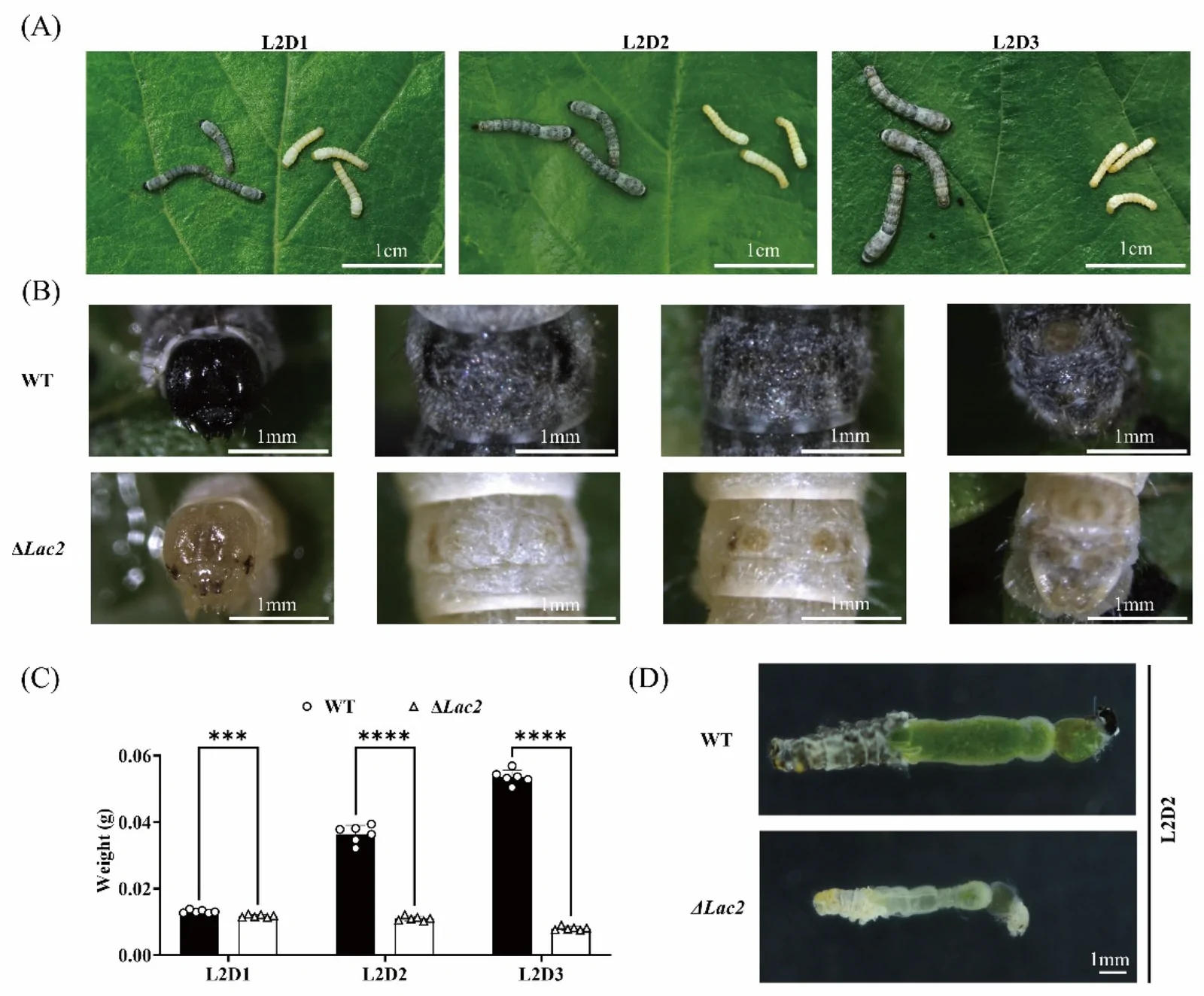
Loss of *BmLac2* resulted in impaired pigmentation and larval developmental arrest. (**A**) Representative images of wild-type (WT) and mutant (Δ*Lac2*) 2nd-instar larvae at each day of the 2nd instar (L2D1-L2D3). (**B**) Enlarged images of different tissues in WT and Δ*Lac2* larvae at L2D1. The entire head, dorsal and caudal markings of wild-type larvae exhibited normal melanin deposition. In contrast, the Δ*Lac2* mutant larvae exhibited reduced pigmentation in all regions. (**C**) Body weight statistics of wild-type (WT) and mutant (Δ*Lac2*) larvae on each day of the 2nd instar, with 5 larvae per group and a total of 6 groups measured (*** *p* < 0.001 and **** *p* < 0.0001). (**D**) Observation of intestinal contents in WT and Δ*Lac2* larvae at L2D2 (after feeding mulberry leaves).

Knockout of the *BmLac2* gene caused grossly observable growth arrest in mutant larvae after molting to the 2nd instar (Figure 2A). Additionally, these larvae showed almost complete lethality on day 3 of the second instar and were unable to enter dormancy for third-instar development. After molting to the 2nd instar, the body color of the mutants began to blanch, and this blanched phenotype was observed in almost the entire head, dorsal and caudal markings, leading to an overall pale body phenotype in the larvae (Figure 2B). Analysis of the daily body weight of larvae during the 2nd instar revealed that, in contrast to the daily weight gain in wild-type larvae, the body weight of the mutants not only failed to increase but even decreased progressively day by day (Figure 2C). Dissection of larvae on the L2D2 instar showed that only a small amount of mulberry leaf tissue was present in the gut of the mutants (Figure 2D). Two possibilities could account for this phenomenon: one was that the mutants exhibited anorexia, and the other was that the mutants were incapable of feeding.

### 3.4. BmLac2 Knockout Only Affected Dopamine Levels During Molting Without Altering Feeding Motivation

**Figure 3:**
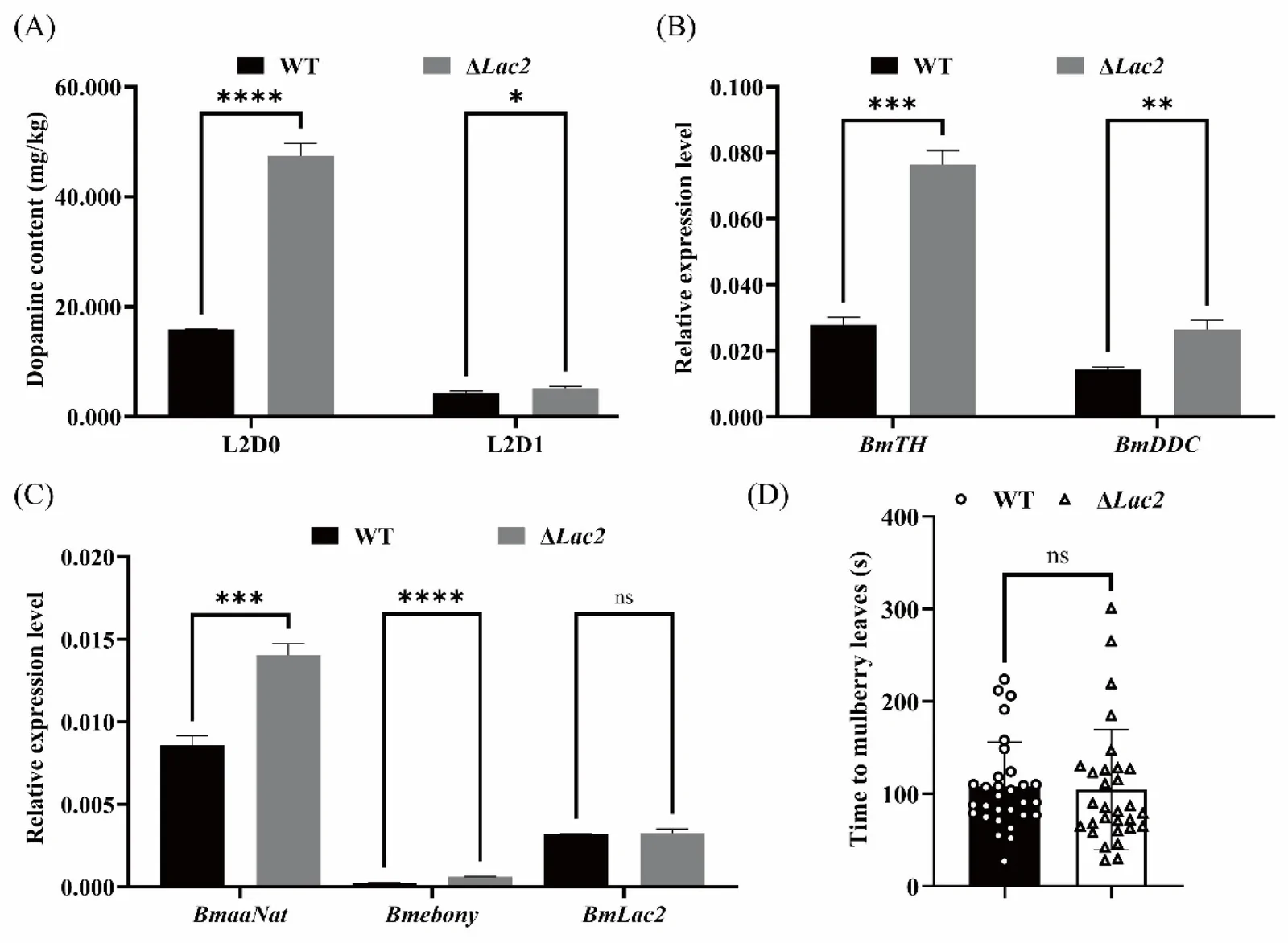
Knockout of *BmLac2* transiently affected dopamine signaling during molting, without impairing feeding motivation. (**A**) Dopamine content in WT and Δ*Lac2* larvae at L2D0 and L2D1. Three larvae were pooled per sample, with three biological replicates. (**B**) The relative expression levels of dopamine synthesis genes *BmTH* and *BmDDC* in WT and Δ*Lac2* larvae at L2D1. (**C**) The relative expression levels of dopamine metabolic genes *BmaaNat*, *Bmebony* and *BmLac2* in WT and Δ*Lac2* larvae at L2D1. Three biological replicates were conducted. Bars represent mean ± standard deviation and statistical comparisons between two groups were performed using two-tailed Student’s *t*-test (* *p* < 0.05, ** *p* < 0.01, *** *p* < 0.001 and **** *p* < 0.0001; ns, non-significant). (**D**) Feeding motivation assay for hungry 2nd-instar larvae using fresh mulberry leaves. The time taken for them to crawl to the leaves and initiate feeding did not differ significantly between groups. N = 30 for each group.

*Lac2* is a key gene involved in dopamine metabolism, and its knockout may affect dopamine levels, thereby altering feeding motivation. Therefore, we further examined whether dopamine levels in *BmLac2* mutant larvae were affected. As expected, knocking out *BmLac2* led to a significant increase in dopamine levels during the dormant stage (L2D0). However, after the completion of ecdysis (L2D1), dopamine levels decreased rapidly and returned to levels similar to those of the wild-type (Figure 3A). In addition, we performed qRT-PCR analysis on the key genes of the dopamine signaling pathway, including two synthetic genes *BmTH* and *BmDDC*, as well as three metabolic genes *BmaaNat*, *Bmebony* and *BmLac2* at L2D1. *BmTH*, *BmDDC*, *BmaaNat*, *and Bmebony* were all upregulated, and the expression level of *BmLac2* was not influenced in the mutant larvae at L2D1 (Figure 3B,C). The elevated dopamine levels during molting in mutants can be attributed to upregulated *BmTH* and *BmDDC* and the deletion of *BmLac2*, while the subsequent return to basal levels after ecdysis is likely driven by increased expression of *BmaaNat* and *Bmebony*, which promote dopamine metabolism. Dopamine has been shown to regulate feeding motivation in various insect species by altering its expression levels. Hence, we performed a feeding motivation assay to examine feeding appetite in the *BmLac2* mutant larvae at L2D1. The result showed that wild-type and mutant larvae spent comparable time initiating feeding, demonstrating no significant difference in feeding desire between them (Figure 3D). The above results indicated that developmental arrest in mutants was likely due to impaired feeding ability rather than reduced feeding motivation regulated by dopamine level.

### 3.5. Deletion of BmLac2 Caused Mandible Development Defects and Severe Impairment of Feeding Behavior

**Figure 4:**
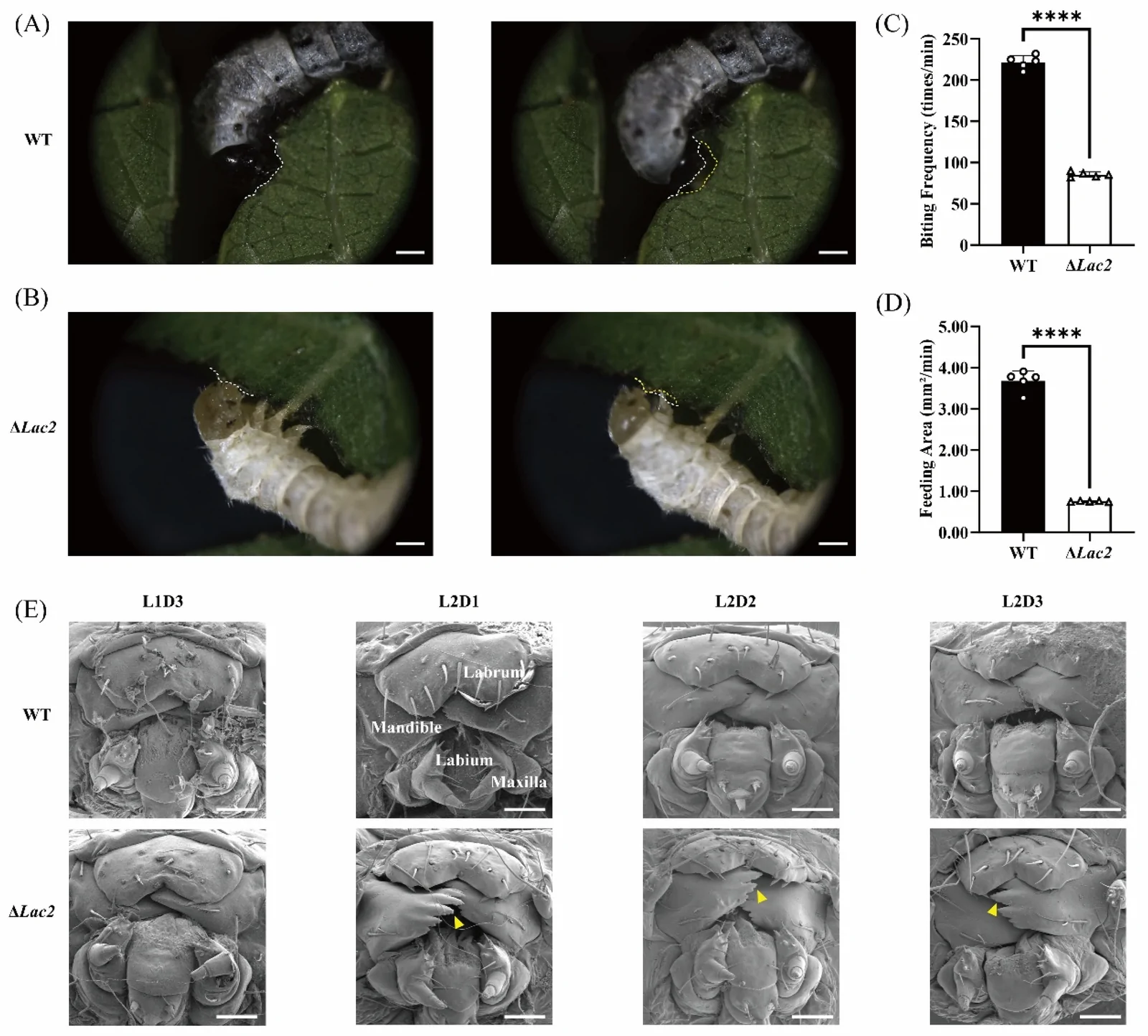
Deletion of *BmLac2* caused mandibular defects and severe impairment of feeding behavior. (**A**) Representative images showed the feeding status of wild-type (WT) larvae at L2D1. White dashed lines indicate the morphology of mulberry leaves before feeding, and yellow dashed lines represent their morphology after 15 s of feeding. (**B**) Representative images showed the feeding status of mutants (Δ*Lac2*) at L2D1. Scale bars:500 μm. (**C**) Quantification of biting frequency in WT and Δ*Lac2* larvae at L2D1 per minute, with three larvae per group and five biological replicates. (**D**) Quantification of mulberry leaf consumption area by larvae per minute, with three larvae per group and five biological replicates (**** *p* < 0.0001). (**E**) Representative scanning electron microscope images of mandible morphology in wild-type (WT) and mutant (Δ*Lac2*) larvae at different stages. Abnormally developed mandibles are indicated by yellow triangles. Scale bars:200 μm.

To determine whether the mutant larvae exhibit feeding impairment, we recorded and compared videos of the first feeding behavior of wild-type and mutant larvae immediately after their 1st ecdysis (Figure 4A,B) (Videos S1 and S2). Upon contact with fresh mulberry leaves, the newly ecdysed silkworms initiated feeding activities rapidly. Within one minute, the feeding frequency of wild-type silkworms on mulberry leaves was approximately 222 times, whereas that of the mutants was only about 85 times, representing a 61.71% reduction compared with the wild type (Figure 4C). In addition, another significant difference was also observed in the feeding area per minute. The wild type reached approximately 3.68 mm^2^/min, while the mutant was merely 0.76 mm^2^/min, a decrease of 79.47% relative to the wild type (Figure 4D). These results confirmed that the developmental arrest of the mutants stems from their inability to feed on mulberry leaves as efficiently as the wild-type silkworms. The mandibles of the silkworm are the core structures for cutting mulberry leaves. Here, we dissected intact silkworm heads and observed the larval mandibles under a scanning electron microscope (SEM). Before the 1st-instar dormant stage (L1D3), the mutants were still able to feed normally, with their mandibles showing a normal structure (Figure 4E). After the 1st-instar dormancy, the mutants lost the ability to feed properly because their mandibles failed to close normally, accompanied by damage to the serrated structures. This structural abnormality was irreversible and persisted until the late 2nd-instar (L2D3). These results demonstrated that developmental arrest in mutants was due to the mandibular defects and abnormal feeding behavior.

### 3.6. RNA-Seq and qRT-PCR Analysis of BmLac2 Knockout

**Figure 5:**
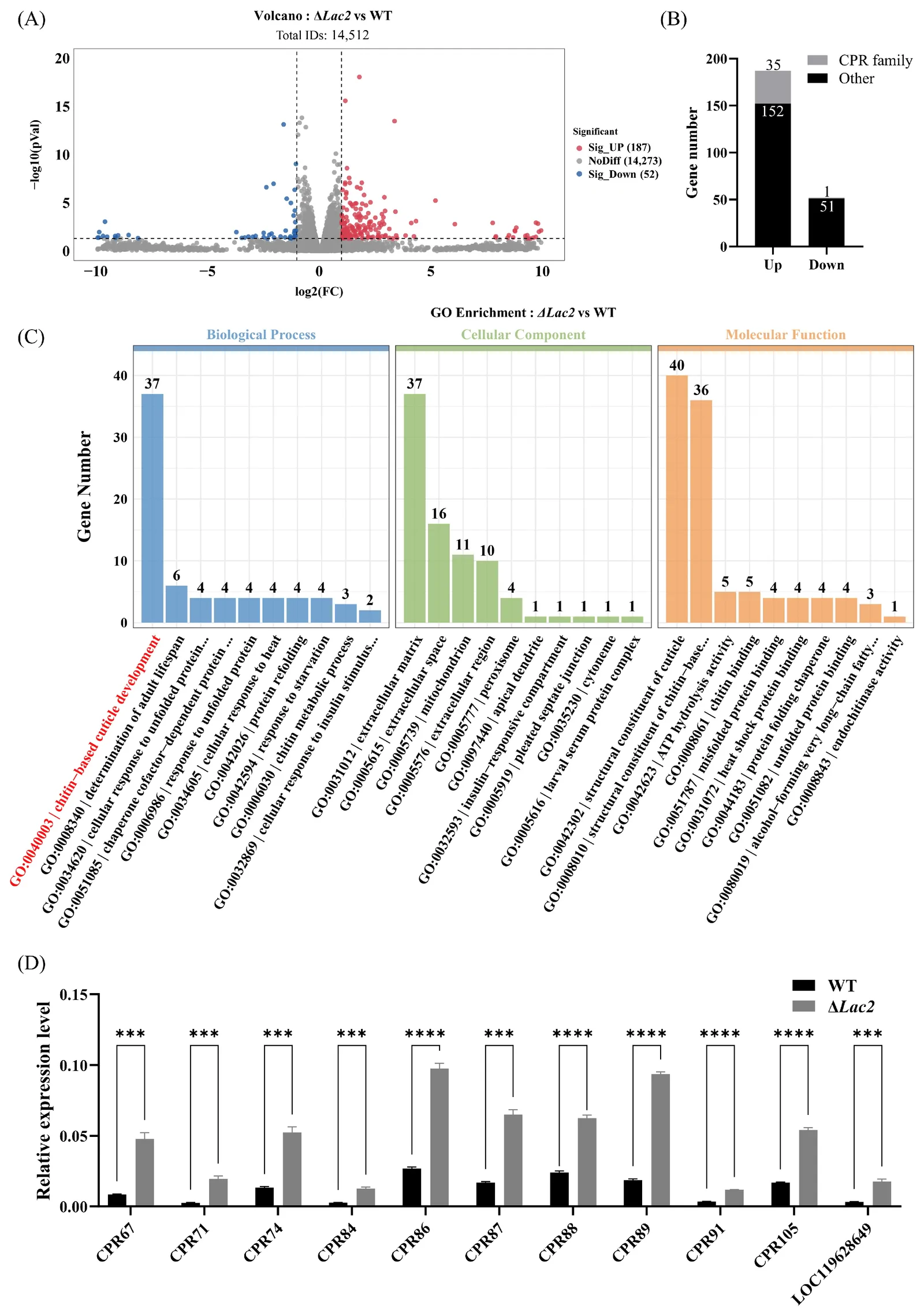
RNA-seq analysis of *BmLac2* mutant larvae relative to the wild-type (WT) larvae. (**A**) Statistical chart of significantly differentially expressed genes (DEGs) between the WT and Δ*Lac2* silkworms at L2D1. The |log2 FC| ≥ 1 and *p*-Value ≤ 0.05 were used as thresholds to identify genes with significant expression differences. (**B**) Counts of CPR family genes among up and down-regulated genes. Gray represents differentially expressed CPR family genes, black represents differentially expressed other genes. (**C**) Gene ontology (GO) enrichment analysis of differentially expressed genes between Δ*Lac2* and WT larvae highlights alterations in the cuticle development and structure pathways. Red text represents the most significantly affected GO terms. (**D**) Verification of several DEGs related to chitin-based cuticle development pathway, structural constituent of cuticle and structural constituent of chitin-based larval cuticle in the WT and Δ*Lac2* silkworms by qRT-PCR. Bars represent mean ± standard deviation and statistical comparisons between two groups were performed using two-tailed Student’s *t*-test (*** *p* < 0.001; **** *p* < 0.0001).

To explore the molecular mechanisms underlying *BmLac2*-mediated melanization and larval mortality, transcriptomic analysis was performed on individuals from both WT and *BmLac2* mutant strains. In total, 239 genes were significantly changed in the *BmLac2* mutant strain. Among these genes, 187 genes were upregulated and 52 genes were downregulated (Figure 5A). Among these significantly differentially expressed genes (DEGs), 36 belonged to the Cuticle Protein with R&R consensus (CPR) family gene, accounting for 15.06% of all DEGs. Of these, 35 CPR family genes were upregulated and one was downregulated (Figure 5B). GO enrichment analysis revealed that the chitin-based cuticle development was significantly affected in the *BmLac2* mutants (Figure 5C). KEGG enrichment analysis revealed that the genes with altered expression levels due to *BmLac2* deletion were also involved in multiple immune signaling pathways, including the FoxO signaling pathway, the mTOR signaling pathway, and the Toll and Imd signaling pathway (Figure S2A). We further confirmed the genes identified by KEGG and GO enrichment analyses using qRT-PCR. The results showed that with the knockout of the *BmLac2* gene, the mRNA levels of genes related to cuticle formation and exoskeleton sclerotization were all upregulated, including *CPR67*, *CPR86*, *CPR89* and other CPR genes (Figure 5D). Since the mandible is a highly sclerotized derivative of the epidermal cuticle, these molecular changes would directly compromise mandibular cuticle integrity. In addition, the mRNA levels of genes related to development and immunity were also significantly upregulated, including *BmBbx-b8*, *catalase*, and the antimicrobial peptide genes *LOC101739536*, *LOC101739681* and *LOC101739958* et al. (Figure S2B–D). These results suggested that in the *BmLac2* mutant, aberrant cuticle sclerotization not only impaired mandibular structure but also compromised cuticular integrity and melanization, thereby reducing physical protection and triggering immune activation, which together contributed to larval mortality.

## 4. Discussion

Although *Lac2* has been extensively studied for its conserved role in cuticle sclerotization and pigmentation across insects, the research on Lepidoptera is limited, and its potential role in feeding regulation remains unexplored. In this study, we employed a binary transgenic CRISPR/Cas9 system to investigate the physiological functions of *B. mori Laccase2* (*BmLac2*). Our results demonstrated that *BmLac2* not only participated in the cuticle tanning and melanization, but also modulated the feeding behavior of silkworm larvae by maintaining the structural and functional stability of their mandibles. These findings provide insights into the physiological functions of the *Lac2* gene, which encodes a key enzyme in the dopamine signaling pathway.

Multiple sequence alignment of *Lac2* sequences revealed that the functional motifs (Cu-oxidase, Cu-oxidase_2 and Cu-oxidase_3) of *BmLac2* shared a high degree of similarity with those of *Lac2* orthologs from other species. Copper-binding domain motifs are highly conserved sequences in *Lac2* across diverse species [32]. Additionally, a C-X-R-X-C motif and a downstream cysteine-rich region, both structural features conserved across all insect Lac2 proteins, are found in the N-terminal region of BmLac2 [33]. Tissue-specific expression analysis at L5D3 stage showed that *BmLac2* was not ubiquitously expressed in silkworms and was only highly detected in the Malpighian tubules, organs associated with insect innate immunity [34]. This expression pattern is consistent with data from silkworm genomic databases and previous research [7,35]. Though the role of *Lac2* in the testis has not been previously reported, dopamine signaling has been implicated in testicular function in mammals and honey bees [36,37]. Given that *Lac2* is a key enzyme in dopamine metabolism, its expression in the silkworm testis suggests a potential role in male reproductive physiology. The low level of *BmLac2* detected in the cuticle at the L5D3 stage can be attributed to its expression being tightly regulated and limited to the molting stage, after which the expression levels rapidly decline. The spatiotemporal expression profile of *BmLac2* suggested that this gene is closely associated with ecdysis, exoskeleton formation and innate immunity in *B. mori*.

In this study, *BmLac2* mutants exhibited normal growth, development and feeding behavior identical to those of wild-type larvae from hatching until the 1st-instar molting. However, after molting to the 2nd-instar, the mutants displayed a melanin-deficient phenotype with a colorless body, consistent with the phenotype observed in other insects following *Lac2* RNAi [33,38]. Notably, the Δ*Lac2* larvae exhibited abnormal feeding behavior and developmental arrest in the 2nd-instar, with a progressive decline in body weight, and ultimately died at the late 2nd-instar with a 100% mortality rate. Unlike previous studies that primarily relied on RNAi-mediated knockdown, which was also limited in lepidopterans, our CRISPR/Cas9-generated knockout mutants enabled the discovery of previously uncharacterized early larval phenotypes [39]. Notably, we uncovered a feeding defect in *BmLac2* mutants during the early larval stages, a phenotype that might be missed in RNAi-based studies focused on later developmental stages. This feeding impairment led to developmental arrest and ultimately early larval mortality in silkworms. These findings not only expand the function of *Lac2* but also position it as a promising target for early intervention in lepidopteran pest control by disrupting larval feeding behavior before significant crop damage occurs.

It is well established that the insect exoskeleton is primarily composed of the integument and head capsule, including mouth appendages such as mandibles and maxillae [40,41,42]. The mandible, the core masticatory structure of the silkworm mouthparts, is located beneath the labrum [43]. As a specialized sclerotized structure secreted by epidermal cells on the ventrolateral sides of the head capsule, it is a modified appendage of the cephalic exoskeleton and also the most highly mineralized and tanned region of the entire silkworm exoskeletal system [44]. In *Ostrinia furnacalis*, larval microinjection of *Lac2* dsRNA results in failure of larval head pupation [45]. In *D. melanogaster*, although *Lac2* had been reported to be involved in the lighter pigmentation of the mouth, no research had revealed its role in the hardening of the mouth. Our CRISPR/Cas9 experiments discovered that *BmLac2* also played a pivotal role in the tanning of the silkworm mandibular exoskeleton. Scanning electron microscopy (SEM) results in our study revealed that after ecdysis, the mandibles of Δ*Lac2* larvae displayed distinct serrated structures, and the paired left and right mandibles failed to close neatly. Such mandibular structural defects were likely a major cause of feeding impairment, which subsequently led to developmental arrest and mortality in silkworms. Although our data indicate that mandibular defects are a primary cause of the feeding impairment and developmental arrest in *BmLac2* mutants, we recognize that other tissues or pathways may also be involved. The transient dopamine changes during molting, though not affecting feeding motivation, could have additional physiological effects. Future tissue-specific knockout studies and expression localization of BmLac2 in the mandible will help clarify its cell-autonomous versus systemic roles. Moreover, dopamine content alone does not fully reflect the complexity of dopamine signaling. Future studies examining dopamine receptor expression and downstream signaling components will be necessary to determine whether and how dopamine signaling contributes to the *BmLac2*-associated phenotypes.

Previous studies revealed that *Lac2* also played a crucial role in the immune responses of various insect species. In *T. castaneum*, *Lac2* knockdown significantly increases susceptibility to the fungus *Metarhizium anisopliae* infection [5]. RNA-seq analysis in our study further revealed significant perturbations in multiple immune-related signaling pathways, including the FoxO, mTOR, Toll and Imd signaling pathways. The altered expression of genes involved in these pathways in *BmLac2* mutants may be associated with physiological stress caused by impaired cuticle integrity and mandibular defects. As the cuticle serves not only as a protective barrier but also as an important interface between insects and their external environment, disruption of cuticle formation may induce compensatory responses and stress-associated transcriptional changes. Therefore, these transcriptomic alterations may reflect adaptive responses to defective mandibular and cuticular development rather than direct regulatory effects of *BmLac2* deficiency. Further studies are required to determine whether *BmLac2* directly influences these signaling pathways or whether their changes are secondary responses to structural abnormalities caused by loss of *BmLac2*. Moreover, *BmLac2* was relatively highly expressed in the Malpighian tubules of silkworm larvae at L5D3. KEGG enrichment analysis revealed significant alterations in metabolic pathway genes in *BmLac2* mutants. Considering that several identified pathways, particularly FoxO and mTOR signaling, are closely associated with metabolic regulation, these metabolic changes may be related to nutritional stress caused by reduced food intake in mutant larvae. Thus, the metabolic alterations observed in *BmLac2* mutants may represent physiological adaptations associated with impaired feeding behavior rather than direct effects of *BmLac2* loss. Although our results suggest that *BmLac2* deficiency influences immune- and metabolism-related transcriptional responses, whether *BmLac2* has additional physiological functions in these processes requires further investigation.

## 5. Conclusions

In conclusion, our findings establish *BmLac2* as a core regulator of cuticle sclerotization and melanization in silkworms, with pleiotropic effects on mandibular morphology, feeding behavior, and cuticular barrier function. Loss of *BmLac2* triggers a cascade of defects, including mandibular malformation, reduced feeding ability, and immune dysregulation, which collectively result in larval mortality. To our knowledge, this study is the first to link the *Lac2* gene to mandibular development in insects. Phylogenetic sequence analysis in our study revealed that *Lac2* proteins are highly conserved across lepidopteran species, suggesting that *Lac2* is a promising target for early intervention against lepidopteran pests.

## Data Availability

The original contributions presented in this study are included in the article/Supplementary Materials. Further inquiries can be directed to the corresponding author.

## Supplementary Materials

The following supporting information can be downloaded at: https://www.mdpi.com/article/10.3390/insects17090893/s1, Table S1: The primers used in this paper. Figure S1: Protein structure prediction, sequence alignment, phylogenetic analysis, and tissue expression pattern of *BmLac2*. Figure S2: Knockout of *BmLac2* also influenced the expression of immune signaling pathway-related genes in the silkworm. Video S1: Wild-type silkworms feed on mulberry leaves efficiently. Video S2: In contrast to wild-type individuals, the mutant silkworms feed on mulberry leaves less efficiently.
