# Supplementary material for: Laccase2-Mediated Mandibular Development Is Crucial for Larval Feeding Behavior in the Silkworm, Bombyx mori
Source: Insects. 2026 Aug 25;17(9):893. doi: 10.3390/insects17090893 (PMC13607879; doi:10.3390/insects17090893)
Supplement: Supplementary file 1 [file insects-17-00893-s001.zip › Supplementary Information.pdf]

Table S1 The primers used in this paper.

Primer name	Primer sequence (5′ to 3′)	Primer purpose
Lac2-F	GGAGCGGCTTGTCAAGTATG	qPCR
Lac2-R	TTTGCCAAATACCGTGCCAA	qPCR
RP49-F	TCCAACAAGAAGACCCGTCA	qPCR
RP49-R	GCTCTTTCCACGATCAGCTT	qPCR
TH-F	CGCGGACAAAGATTACAGGG	qPCR
TH-R	AACGCAGCCTTGTACTCTCT	qPCR
DDC-F	CTTGGACTGCTGTGATGGC	qPCR
DDC-R	CCGGGCTAGCAATCCAAGTA	qPCR
aaNat-F	GACAAACTGCCGGAGACTTG	qPCR
aaNat-R	ATCAGCATACCGAACCCCAA	qPCR
ebony-F	CAATGGATGCGGAGACACTG	qPCR
ebony-R	CCAAACGCAGATCTCTTCGG	qPCR
CPR67-F	ACAATGGCATTCAAGTTCGTAGT	qPCR
CPR67-R	CGGGTCTCGTGTTGACTCTT	qPCR
CPR71-F	CAATGGCCTTCAAGCTCGC	qPCR
CPR71-R	GCTGTACTGAGGATGAGCGT	qPCR
CPR74-F	ATGCAGTCGCCGCCC	qPCR
CPR74-R	CCACAGCGTTGAATCCGTTG	qPCR
CPR84-F	CCGTAGTTCACGCTACTCCC	qPCR
CPR84-R	GGTCGTCAGCAGAGTACGAG	qPCR
CPR86-F	CTACGCCGGTCACGATGAAT	qPCR
CPR86-R	CTACAGCGTTGAAACCGTGG	qPCR
CPR87-F	ACTACGCCTCCCCAGTTG	qPCR
CPR87-R	CTACAGCGTTGAAACCGTGG	qPCR
CPR88-F	AGTGGTCATCATGAATACGCTCA	qPCR
CPR88-R	GTAATGATGGTGGTGGGCGA	qPCR
CPR89-F	GCTCCTCTGGGCCACTACAG	qPCR
CPR89-R	CTACAGCGTTGAAACCGTGG	qPCR
CPR91-F	ATGATCGCTAAAGCCGCAGT	qPCR
CPR91-R	TTATGGTCGCCGGTATGAGG	qPCR
CPR105-F	CTCACTACTCGTCTGCTGGC	qPCR
CPR105-R	AGTCGTATTTAGGGTGAGCATATT	qPCR
LOC119628649-F	AGATGTTTCGCTTTTTGATACCCT	qPCR
LOC119628649-R	GGTGTCTGGGTCGTCCAAAT	qPCR
BmBbx-b8-F	CTGATGTGGTCAGGTGAGGC	qPCR
BmBbx-b8-R	CTATCCGCAGTACGATGCGA	qPCR
LOC101741329-F	TGGCCATTCAACGACAAGGT	qPCR
LOC101741329-R	ATACCGATGGGTCCCGATGT	qPCR
LOC101739536-F	TTGTGTCTTAACGTCCGCGT	qPCR
LOC101739536-R	GCGTACGTTTTAAAGATCATTTTCC	qPCR
LOC101739681-F	TGAATTTTCTATGTGCTCGTCCA	qPCR

LOC101739681-R	CCCCCAATTAATGCTCTCCCA	qPCR
LOC101740092-F	TTGACCCGTTATCTTGCGCT	qPCR
LOC101740092-R	GTCATGCTCAAAGCCAGCAC	qPCR
LOC101739958-F	TTTGGCTGTGAACTCCTCCT	qPCR
LOC101739958-R	GTTTGAGAGGCAATCGTGGC	qPCR
Lac2-ts1-F	GAATAGCGTATCCACTGAAG	Mutation detection
Lac2-ts1-R	CGACAGAGGAAACTCAAAGG	Mutation detection
Lac2-ts2-F	GACGTCACTGTTCATTAGCG	Mutation detection
Lac2-ts2-R	CTACTTGGAGCACCAAGCTC	Mutation detection
HindIII-F	TCGAGGTCGACGGTATCGATAAGGTTATGTAGTACACATTGTTGTA	Plasmid construction
HindIII-R	TTTTCTTGTTATAGATATCAAAAAAAGCACCGACTCGGTG	Plasmid construction
Overlap-F	GCTAGCCATTGACTCCGCGGAGGTTATGTAGTACACATTGTTGTA	Plasmid construction
Overlap-R	CCGCGGAGTCAATGGCTAGCAAAAAAGCACCGACTCGGTG	Plasmid construction
Lac2-F1	GCATACTGGATCCAAGTTCGGTTTTAGAGCTAGAAATAGC	Plasmid construction
Lac2-R1	CGAACTTGGATCCAGTATGCACTTGTAGAGCACGATATTT	Plasmid construction
Lac2-F2	CGAGCAATCCTCTTCTGTCGGTTTTAGAGCTAGAAATAGC	Plasmid construction
Lac2-R2	CGACAGAAGAGGATTGCTCGACTTGTAGAGCACGATATTT	Plasmid construction

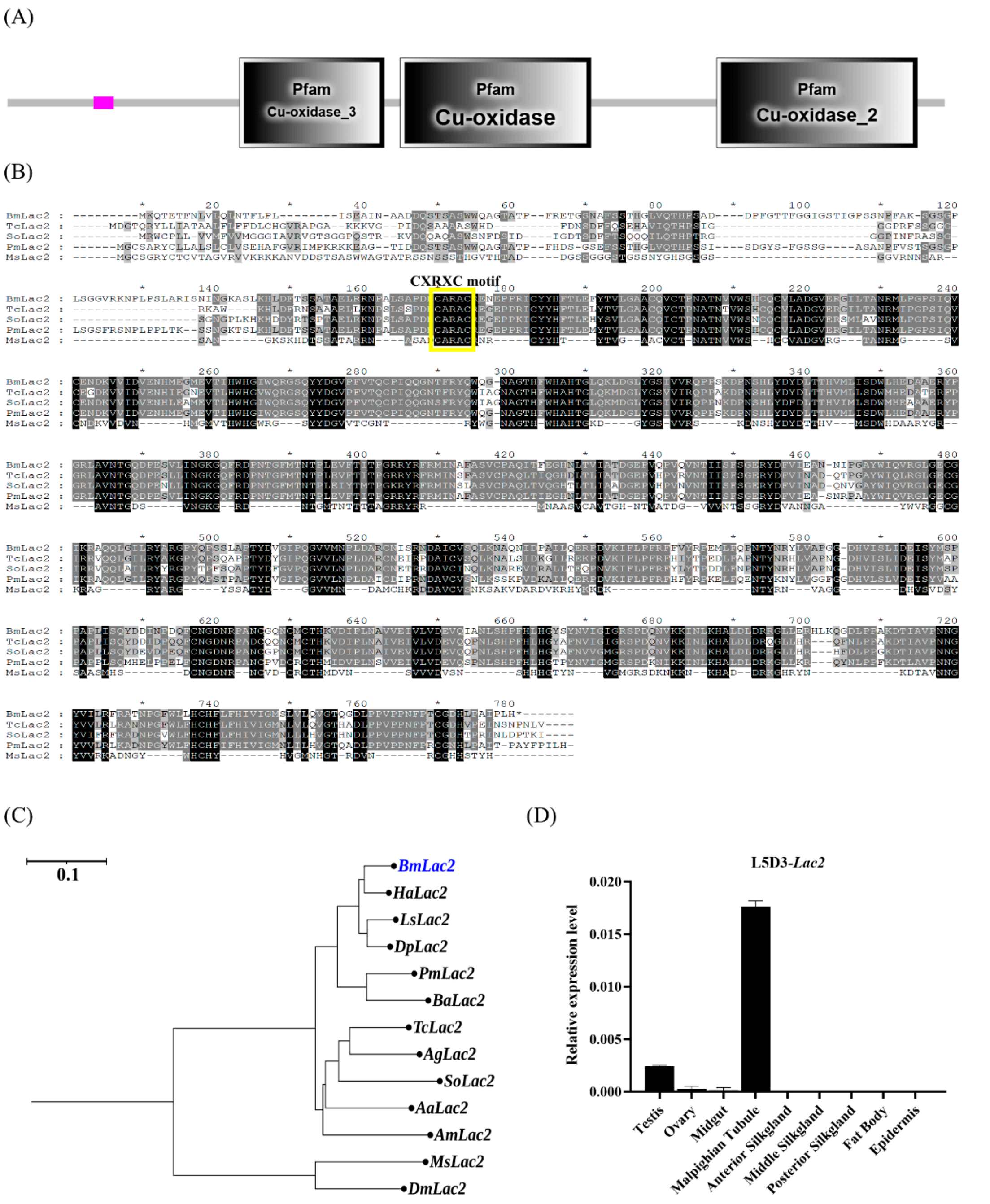

Figure S1. Protein structure prediction, sequence alignment, phylogenetic analysis, and tissue expression pattern of *BmLac2*. (A) Prediction of protein domains was performed using the SMART website (https://smart.embl.de/). Three conserved motifs, Cu-oxidase, Cu-oxidase_2 and Cu-oxidase_3, were present in *BmLac2*. (B) Comparative analysis of Lac2 amino acid sequences across insects and other species. The conserved functional motif, the CXRXC motif, was marked with a yellow box. GenBank accession numbers are as follows: *Bombyx mori* (NP_001103395.1), *Tribolium castaneum* (NP_001034487.2), *Sitophilus oryzae* (XP_030761538.1), *Papilio machaon* (XP_014370419.2), *Manduca sexta* (KAG6448372.1). (C) Maximum likelihood tree of amino acid sequences of Lac2 between *Bombyx mori* and other species. The phylogenetic tree was generated using the MEGAX software, with

Poisson-corrected distances to estimate evolutionary relationships. The branch lengths between protein sequences represent the degree of amino acid divergence, where longer branches correspond to greater differences. A scale bar is provided, with a length of 0.1 denoting a 10% difference in amino acid composition, illustrating the extent of evolutionary divergence. The BmLac2 of *Bombyx mori* was marked with blue. GenBank accession numbers are as follows: *Bombyx mori* (NP_001103395.1), *Helicoverpa armigera* (AHA15412.1), *Leptidea sinapis* (XP_050668955.1), *Danaus plexippus* (XP_061384824.1), *Papilio machaon* (XP_014370419.2), *Bicyclus anynana* (XP_023948182.2), *Tribolium castaneum* (NP_001034487.2), *Anoplophora glabripennis* (XP_018575474.1), *Sitophilus oryzae* (XP_030761538.1), *Aedes aegypti* (XP_058122107.1), *Apis mellifera* (KAG9429887.1), *Manduca sexta* (KAG6448372.1), *Drosophila melanogaster* (NP_724413.2). (D) Tissue expression profile of *BmLac2* from wild-type (WT) at fifth instar day 3 (L5D3) was determined by qRT-PCR.

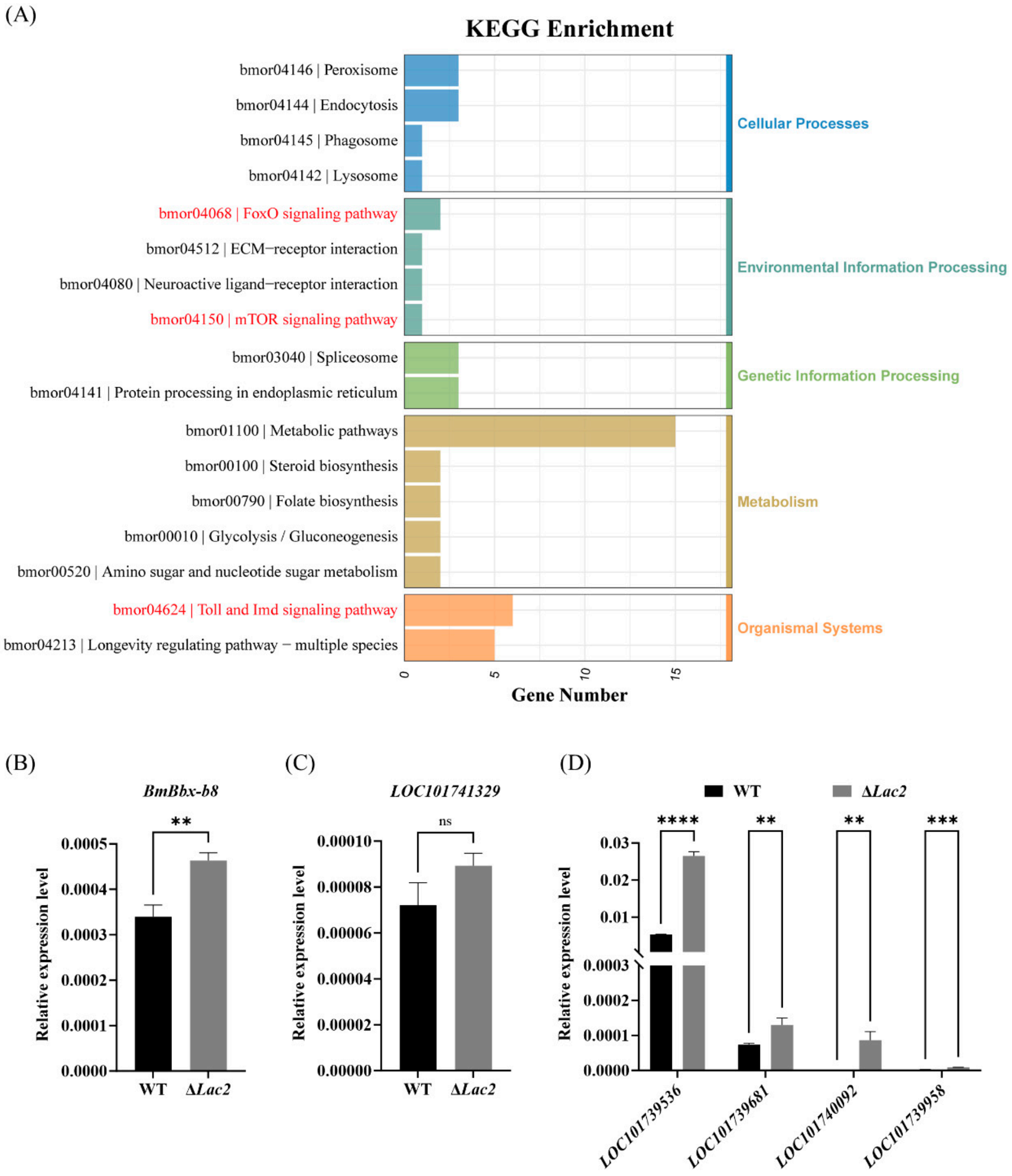

Figure S2. Knockout of *BmLac2* also influenced the expression of immune signaling pathway-related genes in the silkworm. (A) KEGG pathway enrichment analysis of DEGs indicated that compared with the wild-type (WT), immune-related signaling pathways in Δ*Lac2* showed significant alterations, including the FoxO signaling pathway, the mTOR signaling pathway, and the Toll and Imd signaling pathways. (B-D) Six DEGs related to multiple immune signaling pathways were confirmed by qPCR. Asterisks denote significant differences compared with WT, as determined by Student's *t*-test. Significance levels are defined as $**p < 0.01$; $***p < 0.001$; $****p < 0.0001$; ns, non-significant.

Video S1. Wild-type silkworms feed on mulberry leaves efficiently.

Video S2. In contrast to wild-type individuals, the mutant silkworms feed on mulberry leaves less efficiently.
